# Editorial: Updates on innate immune responses in hepatic health and disease

**DOI:** 10.3389/fimmu.2023.1190405

**Published:** 2023-03-28

**Authors:** Hans A. R. Bluyssen, Antonios Chatzigeorgiou

**Affiliations:** ^1^ Laboratory of Human Molecular Genetics, Institute of Molecular Biology and Biotechnology, Faculty of Biology, Adam Mickiewicz University, Poznan, Poland; ^2^ Department of Physiology, Medical School, National and Kapodistrian University of Athens, Athens, Greece

**Keywords:** innate immunity, macrophages, Kupffer cells, innate like T cells (ILCs), macrophage scavenger receptor 1 (MSR1)

The liver, the largest solid organ of the human body, receives more than 80% of its blood supply from the gut through the portal vein and acts as a ‘filter’ for intestinal bacterial products. To effectively and quickly defend against potentially toxic agents without provoking aberrant systemic immune responses, the liver has developed a strong innate immune system (1). Macrophages, both tissue-resident, called Kupffer cells, and infiltrating from the blood, neutrophils, natural killer (NK) cells and innate-like T cells (both NKT cells and Mucosal Associated Invariant T (MAIT) cells) comprise the main innate immune cell populations in the liver (1, 2). Innate immune cells are indispensable for the maintenance of liver homeostasis, while increasing evidence supports their role in liver disease, also beyond infectious diseases (3). Specifically, they may either promote or resolve inflammation, and promote tissue regeneration or fibrosis, showing distinct and contradicting roles in acute liver injury, acute-on-chronic liver injury and chronic liver disease (4).

This special issue is a collection of 1 mini-review, 2 reviews, and 1 research article connected to innate immune responses in Acute liver disease, Acute-on-chronic liver disease and Chronic liver disease.

## Acute liver disease

Deregulation of macrophage balance is critical for the progression or resolution of acute liver injury. The most abundant macrophages in the liver are the tissue-resident macrophages, called Kupffer cells, and monocyte-derived macrophages. In a recent article, Flores Molina et al. examined the spatial distribution and roles of these two cell populations in mouse acute liver injury. The researchers aimed to characterize macrophage responses in mice after CCl4-induced acute liver injury during the early necrotic/inflammatory phase and later repair phases. They identified two main macrophage populations at the site ofinjury which showed distinct kinetics, distribution and morphology: monocyte-derived macrophages (MoMFs) increased dramatically during the necrotic/inflammatory phase,phagocytizing dead hepatocytes and actively secreting proinflammatory cytokines, which affected hepatic stellate cells. On the other hand, tissue-resident IBA1^+^CLEC4F^+^ Kupffer cells (KCs) were reduced during the inflammatory phase, but were restored as the main macrophage population in the repair phase.

## Acute-on-chronic liver disease

Acute-on-chronic liver failure (ACLF), i.e. acute decompensation of a cirrhotic patient due an intra- or extra-hepatic trigger, is a severe condition often complicated by multiorgan failure and high short-term mortality. Khanam and Kottilil summarized the implication of innate immunity in this phenomenon. Indeed, innate immune responses are central for patient prognosis: the magnitude of the innate immune response correlates with clinical disease severity, including the well-established Child-Turcotte-Pugh index a model for end-stage liver disease, and sequential organ failure assessment score. The acute liver injury triggers activation of innate immune cells, which secrete proinflammatory cytokines and chemokines both locally and in the circulation leading to the development of systemic inflammatory response syndrome. Subsequently, a compensatory anti-inflammatory response takes place, leaving patients vulnerable to opportunistic infections. Neutrophils, monocytes/macrophages, dendritic cells, myeloid derived suppressor cells (MDSCs) and natural killer (NK) cells are the main innate immune cells involved in ACLF. Expression of the chemokine receptors CXCR1/CXCR2allows circulating neutrophils to transmigrate into the liver. Hyperactivation of neutrophils leads to the release of proinflammatory cytokines, as well as reactive oxygen species and myeloperoxidase which aggravate tissue damage. Blockades of CXCR1/CXCR2 may hold therapeutic potential in this hyperinflammatory phase (5). NK cells are also excessively activated in the hyperinflammatory phase of ACLF and produce high amounts of TNF-α, TRAIL, and FasL promoting hepatocyte apoptosis. On the other hand, the persistent activation of innate immune cells can lead to their exhaustion. Impaired phagocytic activity of neutrophils may lead to the development of sepsis due to an inability to remove bacteria. Persistent liver injury in ACLF also decreases expression of HLA-DR on monocytes, and upregulates MER receptor tyrosine kinase (MERTK) expression, inhibiting their proinflammatory responses and limiting their ability to control infection (6).

## Chronic liver disease

Activation of macrophages is also central in the progression of chronic liver disease. Non-alcoholic fatty liver disease (NAFLD)isthe most prevalent liver disease, currently affecting approximately 25% of the population worldwide. NAFLD is primarily characterized by fat accumulation in the liver parenchyma, butcan progress to a more severe, inflammatory stage characterized byincreased oxidative stress and hepatocyte damage with/withoutfibrosis, termed non-alcoholic steatohepatitis (NASH)(7).Macrophages are key players in the development and progression of NASH. A review by Sheng et al. focused on Macrophage scavenger receptor 1 (MSR1), which contributes to the phagocytosis of pathogenic microorganisms, but also of modified lipoproteins such as oxidized low density lipoprotein (oxLDL) by macrophages and is increased in mouse models and in patients with NASH. In the early stages of NAFLD, dysregulated lipid metabolism promotes the expression of MSR1 in Kupffer cells and tissue-infiltrating monocyte-derived macrophages (MoDMs). MSR1-mediated oxLDL uptake and limited lipid efflux promote the formation of foam cells by KCs and/or MoDMs and further lipid accumulation in the liver (8). In turn, foam cells release proinflammatory cytokines and aggravate the injury of hepatocytes. Thus, anti-MSR1 blocking antibodies which have been previously shown to inhibit TNF-α, IL-6 and IL-8 production (8) may comprise a valuable therapeutic target in NASH and other inflammatory disorders.

Innate like T cells (ILCs) are a population of lymphocytes that have gained significant attention in the past few years due to their unique combined innate and adaptive immune properties. The most prevalent among ILCs are natural killer T (NKT) and mucosal-associated innate T (MAIT) cells, which express semi-invariant T cell receptors without antigen specificity (9). The liver is enriched in tissue-resident NKT and MAIT cells and their distinct ability to recognize non-peptide antigens, such as lipids and microbial metabolites, renders them potential early sensors of altered liver function. Papanastasatou and Verykokakis provide an overview of their actions during the development of HBV/HCV- or NAFLD/NASH-related hepatocellular carcinoma (HCC). Specifically, during chronic HBV infection, MAIT cells decrease in number in the liver of patients, while NKT cells increase. On the other hand, both MAIT and NKT numbers increase in the liver already from the earliest stages of NAFLD; nevertheless, they decrease later on in patients who develop hepatocellular carcinoma. Delineation of the molecular interactions between ILCs and the surrounding immune cells, as well as of their kinetics in different liver diseases, will foster the understanding of hepatic immune tolerance/disease.

This Research Topic comprises a collection of excellent articles, which provide a valuable update on the contribution of innate immune cells in liver pathophysiology, ranging from acute up to chronic liver diseases. In addition, it offers novel insight in the identification of potential therapeutic targets which can lead to the development of novel treatment strategies.

## Author contributions

All authors listed have made a substantial, direct and intellectual contribution to the work, and approved it for publication.
